# Effectiveness of a recombinant human follicle stimulating hormone on the ovarian follicles, peripheral progesterone, estradiol-17β, and pregnancy rate of dairy cows

**DOI:** 10.14202/vetworld.2016.699-704

**Published:** 2016-07-05

**Authors:** Mohamed Ali, Zeitoun Moustafa M.

**Affiliations:** Department of Animal Production and Breeding, Qassim University, College of Agriculture and Veterinary Medicine, Buraidah 6622, Saudi Arabia

**Keywords:** dairy cows, estradiol-17β, follicles, progesterone, recombinant human follicle stimulating hormone

## Abstract

**Aims:**

This study aimed at elucidating the effects of recombinant human follicle stimulating hormone (r-hFSH) on the ovarian follicular dynamics, progesterone, estradiol-17β profiles, and pregnancy of dairy cows.

**Materials and Methods:**

Three groups (G, n=5 cows) of multiparous dairy cows were used. G1 (C) control cows were given controlled internal drug release (CIDR) and prostaglandin F2α; G2 (L) cows were given low dose (525 IU and G3 (H) cows were given high dose (1800 IU) of r-hFSH on twice daily basis at the last 3 days before CIDR removal. All cows were ultrasonically scanned for follicular growth and dynamics, and blood samples were collected every other day for two consecutive estrus cycles for the determination of estradiol-17β and progesterone.

**Results:**

Estrus was observed in all C and L but not in H cows. Dominant follicle was bigger in L compared to C and H cows. Dominant follicle in C (16.00±2.5 mm) and L cows (17.40±2.3 mm) disappeared at 72 h after CIDR removal. However, in H cows, no ovulation has occurred during 7 days post-CIDR removal. Progesterone was not different (p>0.10) among groups, whereas estradiol-17β revealed significant (p<0.01) reduction in H (15.96±2.5 pg/ml) cows compared to C (112.26±26.1 pg/ml) and L (97.49±15.9 pg/ml) cows. Pregnancy rate was higher in L cows (60%) compared with C cows (20%). However, H cows were not artificially inseminated due to non-ovulation. Only a cow of C group has calved one calf, however, 2 of the L cows gave birth of twins and a cow gave single calf.

**Conclusion:**

Administration of a low dose (525 IU) of r-hFSH resulted in an optimal size of dominant follicle, normal values of progesterone and estradiol-17β, and 40% twinning rate, howeverusing 1800 IU of r-hFSH, have adverse effects on ovarian follicular dynamics and hormonal profiles with non-pregnancy of dairy cows raised under hot climate.

## Introduction

Superovulation of superior cows has been an approach targeting the need for large number of good quality embryos to be transferred [1]. Although much has been done to improve the result of the superovulatory treatment, there are still obstacles facing the purity of hormone, and consequently, the good quality embryos collected from a donor.

Due to the long acting half-life of equine chorionic gonadotrophin (eCG) in the blood of the stimulated females, adverse effects occur on the embryo quality to be eligible for transfer [2]. On the other hand, follicle stimulating hormone (FSH) was found to be more efficient to produce better quality embryos, though it requires to be administered in a series of injections due to its short acting half-life of about 5 h [3]. The preparation of FSH differs from batch to another and also its potency varies according to the species source [4]. Several experiments of superovulation in cattle have been conducted last decades using porcine, bovine, and ovine FSH [1,2].

The preparations to induce superovulation include; eCG derived from pregnant mares (i.e., previously named; pregnant mare’s serum gonadotrophin), extracts of domestic animal pituitaries, particularly those of the pig, ovine, and horse of various degree of purity and FSH to luteinizing hormone (LH) ratios, recombinant bovine somatotrophin combined with FSH; and gonadotrophin of pituitary origin extracted from human postmenopausal urine (human menopausal gonadotrophin).

In this study, we focused on using a new preparation of FSH in cattle, that is, a recombinant human follicle stimulating hormone (r-hFSH) produced in Chinese hamster ovarian cells by recombinant DNA technology [5]. The therapeutic indications for r-hFSH in women are anovulation [including polycystic ovarian syndrome (PCOS)], stimulation of multifollicular development, and severe LH and FSH deficiency. It also causes stimulation of spermatogenesis in men who have congenital or acquired hypogonadotropic hypogonadism with concomitant human chorionic gonadotrophin therapy. Therefore, this study aimed to determine the effect of a low versus high dose of r-hFSH on the follicular dynamics, progesterone, and estradiol-17β profiles, and pregnancy rates in dairy cows raised under hot climate.

## Materials and Methods

### Ethical approval

This study has been approved by the animal rights and ethical use committee of Qassim University.

### Animal’s management and location

This study was conducted at the Experimental Agriculture and Veterinary Research Station, Qassim University, Saudi Arabia, during August 2014-March 2015. 15 Friesian and crossbred multiparous cows (?3-4 years) were selected for the study. These cows were healthy, cycling normally, and non-pregnant as confirmed by transrectal ultrasound palpation. The ovaries were also palpated for the presence of either follicles or an active corpus luteum (CL). Cows were supplemented with commercial concentrates (16% crude protein) at a rate of 8 kg/cow/day. Freshwater and mineral blocks were available *ad libitum*.

### Experimental outline

**Figure-1:**
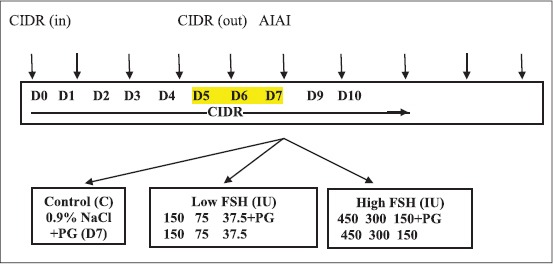
Diagrammatic outline of the experimental design.

All cows were inserted with controlled internal drug release device (CIDR^®^ device; inter-Ag, New Zealand) for 7 days. This device is readily coated with 1.38 g of progestagen in a silicon rubber elastomer. Cows were randomly and equally divided into three groups (control [C], low dose [L], and high dose [H]). Control cows were inserted with CIDR for 7 days and given cloprostenol (500 µg i.m., Estrumate^®^, Schering-Plough Animal Health, USA) at CIDR removal. Low dose cows (L) were inserted with CIDR as control and given r-hFSH (i.m., GONAL-f, MERK, SERONO, Switzerland) in descending pattern on twice daily basis on day 5, 6, and 7 at doses of 300, 150, and 75 IU (i.e., total dose of 525 U), respectively. High dose cows (H) were also inserted with CIDR for 7 days and received FSH (i.m.) on twice daily basis on day 5, 6, and 7 at doses of 900, 600, and 300 IU (i.e., total dose of 1800 U), respectively. All cows in L and H groups were given (i.m.) 500 µg estrumate at the fifth dose of FSH. Figure-1 shows the experimental outline of the experiment.

### Estrus exhibition and insemination

24 h after the conclusion of the treatment, estrus signs were visually observed in all cows in the C and L groups. However, none of the cows of H group exhibited estrus within 7 days post superovulatory treatment. Artificial insemination was performed immediately after estrus exhibition and repeated twice at 12 h interval in all estrus cows. Straws (0.5 ml) of fertile tested bull containing 20 X 10^6^ live sperm were used for inseminating the estrus cows.

### Ultrasound examination

The ovaries were examined by an ultrasound scanner (Pie-medical, Netherlands) with a 5 MHz transrectal probe to determine the time of ovulation and follicular populations. Ovulation occurrence was considered at the day preceding the sudden disappearance of the dominant follicle during examination. For confirmation that ovulation had occurred, the cow was reexamined 24 h later and continuously examined to determine the sizes of follicles on once a week during the subsequent 30 days. Pregnancy was also determined in C and L cows by the ultrasound examination after 30 days of insemination. Cows in H group were scanned for ovulation and follicular dynamics.

### Blood sampling and sera processing

Tail venipuncture blood samples were collected in plain vacutainer^®^ tubes for hormone assay once daily beginning at day of CIDR removal for 3 consecutive days. Thereafter, blood samples were collected once a week during the next 30 days. Blood samples were cooled at 5°C for 2 h and centrifuged (3000 rpm/5°C), sera were harvested and stored deep frozen (- 20°C) until analysis.

### Progesterone (P4) and estradiol 17-β (E2) determinations

EIA Commercial kits (Human, Germany) were used for the determinations of P4 and E2. The sensitivity of progesterone ranges between 0.03 and 0.07 ng/ml. The intra- and inter-assay coefficient of variations for progesterone values were 3.7% and 5.1%, respectively [6]. The sensitivity of estradiol 17-β was 3.6 pg/ml. The intra- and inter-assay coefficient of variations were 3.2% and 5.6%, respectively [7].

### Statistical analysis

Data were analyzed using SPSS statistical software release. Number of cows displayed estrus, inseminated and became pregnant was expressed as a percentage of the total number within a group. Due to the non-normally distributed nature of the data, Kruskal–Wallis one-way ANOVA (non-parametric statistical test) was used to test for the presence of statistically significant difference among three groups. Data for hormones were analyzed by the least square analysis of variance by repeated measures [8]. Proportional data (pregnancy rate and estrus expression) were analyzed by Chi-square.

## Results

### Estrus, conception, and calving

Table-1 shows that percent of the estrus presentation was higher in L (100%) compared with C (60%) cows. Contrariwise, none of the H cows exhibited estrus signs during the 7 days subsequent to CIDR removal, which resulted in non-pregnancy in this group. Administration of a low dose of r-hFSH accelerated (p<0.01) the occurrence of estrus in dairy cows by 22.2 h earlier than that found in control cows. Furthermore, pregnancy rate was 3-folds (60%) in the L cows compared with C (20%) cows. Moreover, two of the pregnant L cows calved twins (one cow calved 2 males and another cow calved a male and female) and the third cow calved a single female. Contrariwise, the only pregnant control cow calved a single male. Mean birth weight for the calf born with twins was 38 kg, however, single calf weighed 42 kg at birth.

**Table-1:** Effect of administration of r-hFSH on the estrus exhibition and pregnancy rate in dairy cows.

Treatment	Number of animals	Number of cows exhibiting estrus (%)	Mean time from CIDR removal to estrus (hours)	Pregnancy rate (%)	Number of calves born
Control (C)	5	3 (60)	75.00±4.0^a^	20 (1)	1
Low FSH (L)	5	5 (100)	52.80±2.9^b^	60 (3)	5
High FSH (H)	5	0 (0)	ND*	0	0

ab Means in the same column with different superscripts are significantly different (p<0.01).

* ND=Not detected, rhFSH=Recombinant human follicle stimulating hormone, CIDR=Controlled internal drug release

### Ovarian follicular dynamics

**Figure-2:**
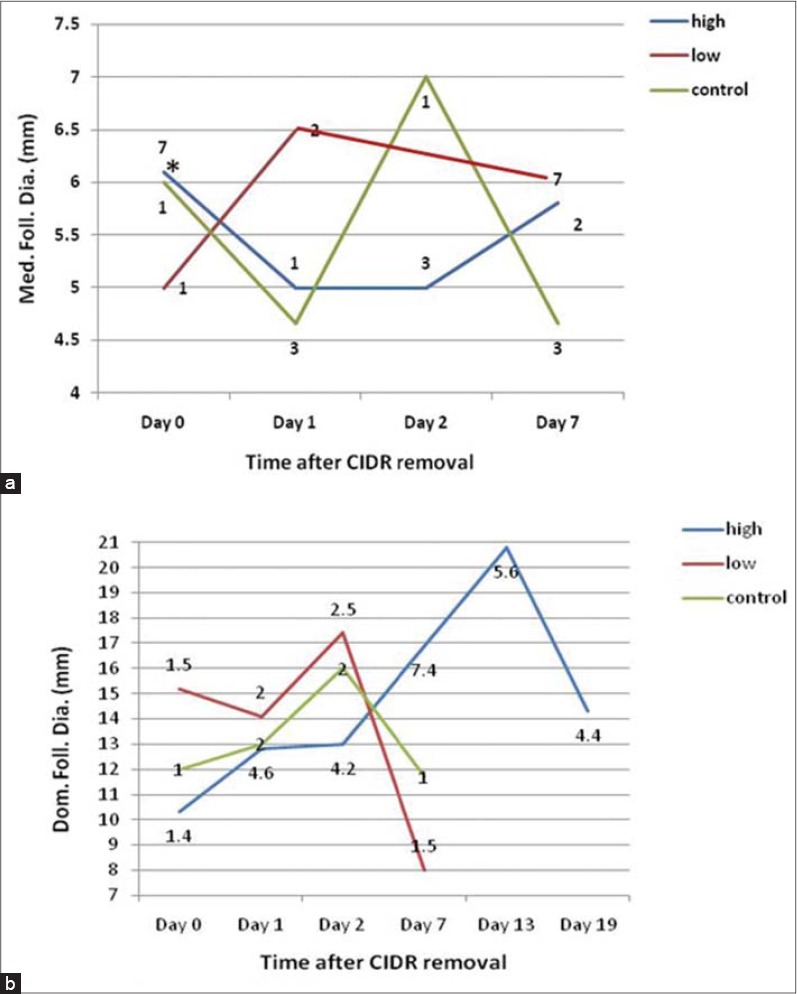
Number of medium (a) and large (b) follicles during days post controlled internal drug release removal in control, low and high-recombinant human follicle stimulating hormone -treated dairy cows.

Figure-2a illustrates number of medium follicles in different treatment groups. The size of medium follicles at CIDR removal was 6.00±0.0, 5.00±0.0, and 6.14±0.4 mm in C, L, and H cows, respectively, with no statistically significant difference. However, seven medium follicles were found in three cows in H group, only one medium follicle was found in each cow in C and L cows. At 24 h post-CIDR removal, mean size of medium follicles in C cows was 4.66±0.33 mm (i.e. total of three follicles in two cows), while size of medium follicles was slightly bigger (6.5±0.5 mm) in two cows of L group. In H cows, only one medium follicle with diameter of 5 mm in one cow was observed.

After 48 h of CIDR withdrawal, no medium follicles were found in L cows, whereas there found one follicle (7 mm) and three follicles (5 mm) per cow in C and H cows, respectively. At day 7 post-CIDR removal, mean number of medium follicles were 1.5, 1, and 3.5 in C, L, and H cows, respectively. Likewise, mean size of medium follicles was 4.66±0.2, 5.66±0.6, and 5.85±0.4 in C, L, and H cows, respectively.

Figure-2b exhibits the population of large follicles throughout the experiment. At day of CIDR removal, number of large follicles was 1, 1.5, and 1.4 in C, L, and H cows, respectively, however, this number was 2, 2, and 4.5 follicles at day 1 and 2, 2.5, and 4.2 follicles at 2 post-CIDR removal in C, L, and H cows, respectively. At day 7, number of large follicles was 1, 1.5, and 7.4 in C, L, and H cows, respectively. Beyond day 7, only H cows were scanned at days 13 and 19 post-CIDR removal and contained decreased large follicles (i.e., 5.6 and 4.4 at day 13 and 19, respectively).

Diameter of large follicles reveals different trends among treatments. The largest follicles at CIDR removal was observed in L cows (15.25±2.6 mm) compared with C (12.0±1.1 mm) and H (10.3±2.2 mm) cows with none significant difference. Near time of ovulation (i.e., around day 2 post-CIDR removal), diameter of largest follicle was 16, 17.4, and 13 mm in C, L, and H cows, respectively, being higher (p<0.05) in C and L than H cows. The ultrasound diagnosis revealed that ovulation has occurred at 90 and 65 h in C and L cows, respectively, post-CIDR removal, however, none of the H cows presented estrus or ovulation during 14 days post-CIDR removal. At day 7, the diameter of largest follicle was larger (p<0.05) in H (16.9±1.8 mm) cows compared with C (11.75±2.1 mm) and L (8.0±0.01 mm) cows. In H cows, the largest follicle approached 20.8 mm at day 13 and regressed to 14.3 mm at day 19. These H cows were subsequently recycled with gonadotropin-releasing hormone for regular cycling which obviously done after about 50 days of CIDR removal.

### Blood progesterone and estradiol-17β

**Figure-3:**
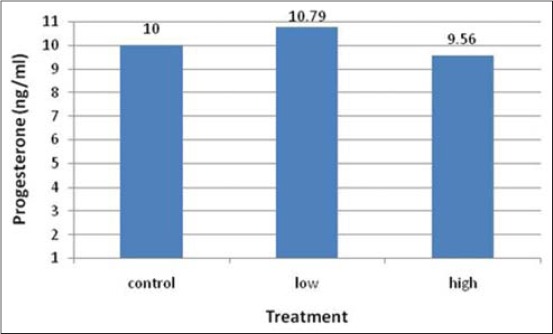
Blood progesterone level in control, low and high-recombinant human follicle stimulating hormone-treated cows.

**Figure-4:**
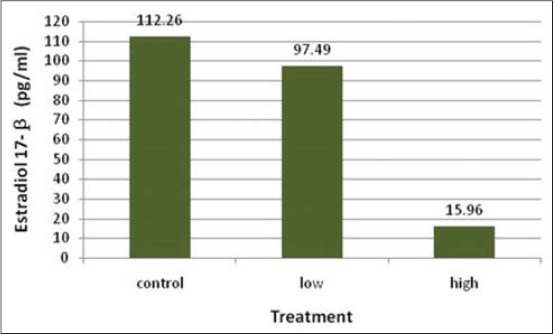
Blood estradiol 17-β level in control, low and high-recombinant human follicle stimulating hormone-treated cows.

Progesterone in blood circulation revealed mean values of 10, 10.79, and 9.56 ng/ml (Figure-3) being none significant (p>0.10) among treatments. On the other hand, estradiol-17β (Figure-4) revealed a significant (p<0.05) decline in H cows (15.96 pg/ml) compared with C (112.26 pg/ml) cows, which was not different than L (97.49 pg/ml) cows.

## Discussion

Since FSH has shown good superovulatory responses accompanied with better number of good quality transferable embryos compared with eCG in gilts [9], deer [10], beef cows [2], goats [11], and dairy cows [12]. Administration of descending dose of FSH has shown acceptable superovulation in dairy cows. The use of bovine, porcine, and ovine FSH has been a common practice in the superovulation regimes in dairy cattle [13]. However, implementing hFSH in the superovulatory programs of dairy cows has not been attempted before. Therefore, the current study focused to examine the use of r-hFSH. It has been found that hFSH is capable of promoting the activation of primordial follicles and maintaining the ultrastructural integrity of caprine preantral follicles cultured *in vitro* for 7 days [14].

The homology of the α-subunit of the hFSH and bovine FSH molecule was found to be 75% [15]. Positions of cysteine residues are highly conserved among species, implying that the disulfide bridges within the α-subunit are identical among α-subunits of different species. Furthermore, it has been reported that the nature of the carbohydrate residues that distinguish the α-subunits of the various gonadotrophins within a species is of great importance [16]. Moreover, as the β-subunit determines the biological specificity of the gonadotrophin [15], it is often referred to as the hormone-specific subunit. For example, the bovine hormones were found to contain very little sulfated (S) and sialylated (N) (0-2% of total oligosaccharides) linkages, whereas human and ovine hormones have relatively large amounts of S-N (10% of ovine FSH and 23% of human LH oligosaccharides are of the S-N type). However, bovine, ovine, and human pituitary glycoprotein hormones display a very similar spectrum of sulfated oligosaccharides [17].

The species-specific differences in the molecular structure of gonadotrophin are well known, even though there is still certain percent of homology among species [18]. It is well known that granulosa cells in female ovaries and sertoli cells in male testicles are the target cells for FSH expressing its specific receptors [19].

Application of recombinant technology has allowed the engineering of a variety of analogs with distinct biological features and therapeutic potential [18]. Several researchers confirmed the interspecies FSH interaction to stimulate folliculogenesis and steroidogenesis [20].

The administration of an excess dose of FSH (1800 IU equivalent to 132 µg) in the current study not only resulted in the disappearance of estrus signs but also impeded the rupture of large follicles (anovulatory response) leading to a PCOS. Apparently, this high dose compromised the hypothalamo-pituitary-ovarian axis leading to a sharp decline in estradiol-17β secretion. The existence of PCOS in H cows might be attributed to the hormonal imbalance occurred in cows [21]. Kanitz *et al*. [22] found that using excessive dosages of FSH can disturb the ovulation process at two levels; at the level of pituitary and the ovarian level. This means that these high doses of FSH might suppress or decrease the LH surge leading to a decreased and/or impediment of the ovulation of large follicles. Another cause for the reduced ovulation rate, in this case, might be attributed to the down regulation of follicular FSH receptors by high doses of homologous ligand. Therefore, each FSH product has an optimal dose range [21].

Various pharmaceutical products with gonadotrophin activity and even various batches within a product can differ in their bioactivity and/or immunoactivity [23]. The differences in the bioactivity of various species-derived FSH were mainly ascribed to the degree of glycosylation of the molecule and to the occurrence of isoforms [24].

The sharp decrease in estradiol-17β levels accompanying the high FSH was previously reported *in vitro* [25] and *in vivo* [26]. The decreased E2 in H cows are most likely a result to the negative feedback of the excess FSH on the hypothalamo-pituitary-ovarian axis [27]. Concurrently, the P4 level in H cows revealed similar value to C and L cows. The cystic follicles in H cows were ultrasonically diagnosed as luteinized follicles, leading to compensation of the inefficiency of the original CL. The presence of luteinized follicles might interpret the lack of E2 secretion by granulosa cells, which are replaced by lutein cells [18]. Contrariwise, administration of a low dose of r-hFSH (525 IU=38.5 µg) resulted in higher than control response of estrus exhibition (100% vs. 60%) and pregnancy rate (60% vs. 20%). Cows given low FSH delivered two sets of healthy twins with an average birth weight of 38 kg/calf.

## Conclusion

Using r-hFSH in stimulating dairy cows during the hot climate was as efficient as using other FSH products derived from bovine, ovine, and porcine pituitaries. The use of a low dosage (525 IU equivalent to 38.5 µg) of r-hFSH in dairy cows would be considered a practical regime to stimulate the inactivity of the ovaries of dairy cows during hot summer months. Moreover, this low dosage of r-hFSH resulted in an increased outcome of born calves (twinning). Much interest must be further paid to the best timing during ovarian follicular dynamics for the FSH to be administered.

## Authors’ Contributions

MA has managed the cows, applied treatments, observed estrus, scanned ovarian structures, neonatal care, and weighing and analyzed data. MMZ has accomplished hormonal analyses, helped in the ultrasound measurements, tabulated the data, and wrote and edited the manuscript. Both authors read and approved the final manuscript.
